# Individual differences among older adults with mild and moderate dementia in social and emotional loneliness and their associations with cognitive and psychological functioning

**DOI:** 10.1186/s12877-022-03517-2

**Published:** 2022-11-15

**Authors:** Elena Carbone, Federica Piras, Francesca Ferrari Pellegrini, Paolo Caffarra, Erika Borella

**Affiliations:** 1 Department of General Psychology, University of Padova, Via Venezia 8, 35131 Padova, Italy; 2 Neuropsychiatry Laboratory, Clinical and Behavioral Neurology Department, IRCCS Santa Lucia Foundation, via Ardeatina 306, 00179 Rome, Italy; 3 Geriatric Clinic Unit, Medical Geriatric Rehabilitative Department, University Hospital of Parma, via Gramsci 14, 43126 Parma, Italy; 4 Section of Neuroscience, University of Parma, via Gramsci 14, 43126 Parma, Italy

**Keywords:** Social and emotional loneliness, Language skills, Behavioral/psychological symptoms, Quality of life, Dementia severity

## Abstract

**Background:**

Loneliness is a major health issue among older adults. The aim of this study was to assess the relationship between loneliness, in its social and emotional facets, and the cognitive (language), and behavioral/psychological functioning as well as quality of life (QoL) in people with mild and moderate dementia, i.e., considering dementia severity as an individual characteristic.

**Methods:**

This cross-sectional study involved 58 people with mild dementia and 55 people with moderate dementia. Participants completed the Social and Emotional Loneliness scale, along with measures assessing their language skills, the frequency and severity of their behavioral and psychological symptoms, and their QoL.

**Results:**

Socio-demographic characteristics and depression, but not loneliness or its social and emotional facets, contributed to explain participants’ behavioral and psychological symptoms, regardless of dementia severity. Loneliness explained, though to a small extent (8% of variance), language skills in people with moderate dementia, with social loneliness only accounting for language skills (18% of variance) in this group. Loneliness also modestly accounted for dysphoria symptoms in both the mildly and moderately impaired (6% and 5% of variance, respectively) individuals with social loneliness predicting dysphoric mood in the former group only (7% of variance). Loneliness also explained, to a larger extent, QoL in both the mildly impaired and moderately impaired individuals (27% and 20% of variance, respectively), its social facet predicting QoL in the mildly impaired (30% of variance), and its emotional facet in the moderately impaired (21% of variance) group.

**Conclusion:**

These findings suggest that loneliness and its facets have a clear impact on perceived QoL, and influence the language skills and dysphoria symptoms of people with dementia, to a degree that depends on dementia severity. The assessment of loneliness and its facets in people with dementia considering dementia severity, and the promotion of social inclusion to reduce it should be considered by professionals.

**Supplementary Information:**

The online version contains supplementary material available at 10.1186/s12877-022-03517-2.

## Introduction

Loneliness represents a major public health issue among older adults [1]: evidence is emerging that loneliness is related to poor health and quality of life (QoL) [2, 3], premature mortality [4], worse cognitive functioning and a greater risk of developing dementia [5, 6], and depression [7, 8]. Though often confused with social isolation, it is now well documented and accepted that the two concepts are distinct. Social isolation is an objective social situation characterized by a lack of relationships with others [9]; loneliness is the subjective, negative feeling of a lack or loss of relationships that results from a perceived mismatch between desired and actual social connections [10, 11], and can thus be present even when individuals have a social network. Loneliness and social isolation are established risk factors for dementia, and their elimination later in life may determine a 4% decrease in dementia prevalence [12], a greater reduction than combatting physical inactivity later in life (2%), and hypertension in mid-life.

Alongside stress-related or biophysiological processes, behavioral and lifestyle factors (e.g., reduced physical activity and mobility, smoking, and scarce financial resources, which are risk factors for cardiovascular disease and consequently, for dementia), as well as psychosocial factors, are among the mechanisms advocated to explain the association between loneliness and cognitive deterioration [13, 14]. For instance, qualitatively impoverished family and friendship relationships and limited social connection (also as a result of stereotyping, prejudice, and/or discrimination against older adults [15], and self-efficacy beliefs [16]), with a resulting diminished cognitive stimulation, have been shown to underlie the relationship between loneliness and cognitive decline [14]. Such diminution in cognitive engagement, as a consequence of a reduction in the number and quality of social interactions, may potentially lead to a greater vulnerability to age-related neuropathological changes, thus heightening the risk of cognitive decline or deterioration [14].

However, the dimensionality of loneliness remains elusive [17, 18]: while some researchers consider it to be a unitary construct [11], others have depicted loneliness as a multidimensional one. Among various conceptualizations, a distinction has been proposed between a social facet of loneliness, which refers to the feeling of missing a wider social network, and an emotional one, which refers to the perceived absence of a close and intimate relationship [19]. These two facets of loneliness have been shown to be only moderately correlated [20], and the few studies that have examined the relationship between them and cognitive/psychological functioning in normal aging suggest different associations. For example, less social loneliness, unlike emotional loneliness, was found associated with better performance on a cognitive measure of executive functioning (semantic fluency) [21]. Emotional and social facets of loneliness are also differently related with health outcomes. Emotional loneliness, but not social isolation or perceived social support, is associated with a greater risk of developing dementia [22], although perceived social support seems to protect against dementia in men [23]. Researchers have found social loneliness, but not the emotional facet, to be associated with longevity in women [24], whereas emotional loneliness is associated with an increased risk of all-cause mortality in older adults living alone [25]. Some evidence suggests that emotional loneliness, and not the perceived quantity of interpersonal connections, is more likely associated with the risk of depression and poor mental health (psychological distress) [26, 27]. In contrast, both facets of loneliness seem to be reciprocally associated with depression [28–30]. This initial evidence seems therefore not only to confirm the impact of loneliness on older adults’ cognitive and psychological functioning, but also supports the conceptual separation between social and emotional facets of loneliness and the importance of considering them separately [20, 31].

Notwithstanding the growing number of studies examining such a major public health problem among the older adult population, little is presently known about whether the latter is associated with cognitive functioning, behavioral and psychological symptoms –BPS– and the QoL of the more vulnerable elderly, such as people with dementia [32, 33]. Despite difficulties in ascertaining whether people with cognitive impairment can accurately evaluate their loneliness [33–35], individuals with dementia report feeling lonelier than older adults without dementia [33–37]. The few studies exploring potential associations between loneliness and general cognitive functioning, BPS and QoL in people with dementia have found loneliness to be related to depressive symptoms [38, 39] and QoL [38, 41], but not to general cognitive functioning [38–41]. A few studies have focused on the association between loneliness and the frequency of BPS characteristics of dementia [39, 40]. However, no associations have emerged between loneliness and the frequency of BPS [40], although some evidence has pointed to loneliness being related to the frequency of certain psychotic symptoms (delusions, hallucinations) [39].

Surprisingly, no researchers have yet attempted to understand whether the social and emotional facets of loneliness are associated differently with cognitive functioning, BPS and QoL outcomes in people with dementia. However, feelings of social loneliness are suggested to increase, and emotional loneliness to decrease with reduced cognitive functioning in people with dementia [34]. Furthermore, at least to our knowledge, no study has considered the role of the dementia stage or severity as an individual characteristic when examining loneliness and its facets. As dementia is a progressive disorder [42], with mildly-impaired and moderately-impaired people characterized by different cognitive and BP profiles, the decrease or lack of social networks is more pronounced as the disease progresses [43], although individuals with less compromised cognitive functions more distinctly perceive the loss of social support and integration [44]. The stigma, fear and misunderstanding surrounding dementia, and the increasing difficulties in interacting with others as dementia-related cognitive, behavioral and psychological symptoms progress also contribute to the observed negative gradient in social relationships [15]. Therefore, loneliness, and its social and emotional facets, might affect people with dementia differently, depending on the stage/severity of dementia, while an increasingly “impoverished environment” -in terms of social contacts and opportunities for social stimulation- might further exacerbate cognitive and BP symptoms in people with dementia, and additionally heighten emotional loneliness and feelings of social exclusion.

In the present study, we thus aimed to explore further the relationship between loneliness and cognitive functioning, BPS, and QoL in people with dementia. In particular, to capture the role of loneliness better, its dimensions (emotional and social) were specifically examined, and the severity of dementia (mild versus moderate) was considered separately as individual characteristic.

Two groups of people with dementia —one mildly and the other moderately impaired—completed a widely-used measure for assessing the general feeling of loneliness, and its social and emotional facets [45, 46]. A specific cognitive domain, language skills [47], given their importance to effective communication and the quality of interpersonal relationships, was innovatively assessed. We also recorded the frequency and severity of participants’ BPS, considered as a whole and with respect to specific behavioral (e.g., agitation, disinhibition, motor and sleep disturbances) and psychological (e.g., anxiety, apathy, dysphoria) symptoms, as assessed by the NeuroPsychiatric Inventory (NPI) [48], and their perceived QoL [49].

Due to the progressive nature of dementia [42], in line with the literature [50], we expected the mildly impaired group, compared to the moderately impaired group, to be younger, with better-preserved language skills, to exhibit worse BPS [51], and to report a higher QoL [52]. We specifically explored whether the mildly-impaired individuals would experience lower loneliness than the moderately impaired [34], as progressing behavioral symptoms and affective/emotional dysregulation may heighten emotional loneliness and social exclusion [30], or whether the opposite pattern would occur, given that the former would be more aware of social stigma and more adversely affected by social isolation.

As previous evidence found no relationship between global loneliness and general cognition in people with dementia [38–41], no association between loneliness and language skills was expected. However, as loneliness could be considered a consequence of the deteriorating social skills that are part of the behavioral, psychological and cognitive changes as dementia progresses [35], a significant association with the language skills needed to engage in meaningful interpersonal relationships could also be expected. Additionally, poor and decreased social contacts and opportunities for social stimulation may further exacerbate the feelings of loneliness and make people with dementia more vulnerable to its negative fallout on their cognitive functioning [37, 53], particularly the language skills needed to reciprocate interpersonal relationships.

Previous evidence [39] pointed to associations between loneliness and specific neuropsychiatric (e.g., psychotic) symptoms, that could thus be expected. An association between loneliness and QoL could be expected as well [39–41] as a supportive social network is known to be a factor promoting QoL in PwD [54, 55].

We also explored whether different associations between the facets of loneliness and the outcomes of interest might emerge as a function of dementia severity, examined here as an individual characteristic, which have not yet considered.

Furthermore, given that experimental evidence suggests that loneliness and depression—although associated—operate as different pathways through which an individual’s cognitive and psychological health is affected [14], we evaluated the potential additional contribution of loneliness besides that of depression on the outcomes of interest. The role of socio-demographic characteristics, as they might influence our outcomes [37, 40, 42, 56], was also examined.

## Materials and methods

### Participants

This cross-sectional study involved 58 mildly-impaired people with dementia (with Mini-Mental State Examination -MMSE- scores, corrected for age and education, of 20–24; M_age_=79.50; SD = 9.21; M_education_=7.38; SD = 3.64; 38 females) and 55 moderately-impaired people with dementia (MMSE scores 14–19; M_age_=87.18; SD = 6.22; M_education_=5.07; DS = 2.26; 40 females) [57, 58] who had taken part in a previous clinical trial aimed at assessing the benefits of the Italian adaptation of the Cognitive Stimulation Therapy in people with mild-to-moderate dementia [59]. Participants were recruited through 16 Italian residential care homes or day centers (14 in northern and two in central-southern Italy) between 2014 and 2019. Inclusion criteria were the following: (a) a diagnosis of major neurocognitive disorder (of any etiological subtype) according to the fifth edition of the Diagnostic and Statistical Manual of Mental Disorders in the mild-to-moderate range (MMSE score ≥ 14); (b) a Clinical Dementia Rating score of 1 or 2 [60]; (c) a satisfactory ability to understand and communicate; (d) no neurodevelopmental disorders, premorbid intellectual disabilities, or current physical illness/disability reported in patients’ clinical documents that might interfere with their participation; and (f) no diagnosed comorbid psychiatric disorders. These criteria ensured participants to be able to comply with all the phases of the trial (i.e., the assessment sessions and the treatment/control activities).

People with mild dementia were younger (*t*_(111)_=-5.46; *p* < .001) and better educated (*t*_(111)_ = 4.07; *p* < .001) than moderately-impaired ones, while the two groups did not differ in terms of gender distribution (χ²_(1,111)_ = 0.68; *p* = .41).

### Materials

#### Loneliness

*Social and Emotional Loneliness scale* (adapted from [45], see [46]). There are six items for assessing loneliness and its emotional and social facets, rated on a Likert scale from 1 (absolutely true) to 5 (absolutely not true). The scale displayed a good-acceptable reliability (in the present sample, total score: alpha = 0.79; Emotional Loneliness subscale: alpha = .64 1; Social Loneliness subscale: alpha = 0.83). The dependent variables were the sum of the scores for: all six items (total Loneliness score), the three for emotional loneliness (Emotional Loneliness subscale) and the three for social loneliness (Social Loneliness subscale). Higher scores indicated more loneliness.

#### Language skills

*Narrative Language Test* (NLT) [47]. This test examines textual competence and discourse information content, assessing narrative abilities in terms of the effective communication of information. Participants are asked to describe a single figure (the “Picnic” picture [61]), and then sets of figures (two cartoon sequences [62]). Descriptions are recorded, transcribed verbatim, and segmented using correct information unit analysis [62], followed by a quantitative textual analysis [63]. Each stimulus has a series of concepts that provide the backbone of the plots which have been identified with a methodology described in [64]. The dependent variable was the sum of correctly and accurately reported concepts, with higher scores indicating a better ability to derive conceptual and informational content from the stimulus.

#### Depression

*Cornell scale* (Cornell) [65]. There are 19 items assessing signs and symptoms of major depression in people with dementia. Each item is rated for severity on a scale from 0 (absent) to 2 (severe). The dependent variable was the sum of the scores for the 19 items. Higher scores indicated worse depression.

#### Behavioral and psychological symptoms

*NeuroPsychiatric Inventory* (NPI) [48]. This tool assesses 12 BPS in dementia patients, that is delirium, hallucinations, agitation, dysphoria, anxiety, euphoria, apathy, disinhibition, irritability, motor disturbances, sleep disturbances, food issues. The dependent variables were the frequency x severity scores on each symptom. Higher scores indicated more frequent and severe symptoms.

#### Quality of life

*Quality of Life - Alzheimer’s Disease scale* (QoL-AD) [49]. There are 13 items assessing subjective components of QoL (e.g., psychological wellbeing) and objective components (e.g., behavioral competence and environment). Each item is rated by participants on a 4-point scale from 1 (poor) to 4 (excellent). The dependent variable was the sum of all the items. Higher scores indicated a better QoL.

### Procedure

For the purposes of the present study, only scores derived at pre-test -that is prior to the completion of the clinical trial- were considered.

The outcome measures here selected were acquired in the following order: the MMSE, the Cornell (session 1); the NPI, the Social and Emotional Loneliness scale (not considered as an outcome variable for the clinical trial), the NLT and the QoL-AD (session 2).

### Statistical analyses

The analyses were conducted using IBM SPSS (IBM, 28th edition, Chicago, IL).

First, group differences in the measures of interest were examined with independent *t*-tests.

To examine the influence of loneliness (and its social and emotional facets), over and above the impact of demographics and depression, on the language skills, behavioral and psychological functioning and QoL of people with mild or moderate dementia, first correlations and then hierarchical regression analyses were run separately for the two groups. In a first set of regression analyses, demographics (age, education, gender) and depression (Cornell) were entered in Steps 1 and 2, respectively, and the total Loneliness score in Step 3, as predictors of the scores for the language skills (NLT), BPS (NPI total score and subscales), and QoL (QoL-AD). Then, in a second series of regression analyses, the Social and Emotional Loneliness subscales were used in Step 3 as predictors of each measure of interest. All models were checked for outliers (Cook’s distance < 1).

A power analysis (G*Power) showed that 58 and 55 participants sufficed to obtain a power of 0.80 to 0.77 with 5 predictors and 0.78 to 0.74 with 6 predictors, respectively, in linear multiple regression analyses, with a medium effect size (f^2^ = 0.25) and a significance level of α = 0.05.

### Results

Table 1 shows the descriptive statistics of the measures of interest by dementia severity, and results from independent *t*-tests. The latter showed that people with mild dementia scored higher for language skills, depression, and QoL (see Table 1). They had more frequent and severe BPS -apart from delirium, euphoria, disinhibition- (see Table 1), and slightly experienced less social loneliness than the moderately-impaired people with dementia (see Table 1). The two groups did not differ in terms of general loneliness or its emotional facet (see Table 1).

**Table 1:** Descriptive statistics of sample’s demographics and measures of interest, by dementia severity, and results of independent t-tests on the differences between the two groups for each outcome of interest

	Mild dementia severity (N = 58)		Moderate dementia severity (N = 55)		Independent t-test results	
	*M*	*SD*	*M*	*SD*	t_(111)_	*p*
Narrative Language Test	12.72	4.99	8.91	4.17	4.39	< 0.001
Cornell scale	6.40	6.09	2.85	3.30	3.81	< 0.001
NPI (total score)	13.72	15.47	4.20	5.64	4.30	< 0.001
*NPI- delirium*	0.14	0.63	0.36	0.82	-1.36	0.10
*NPI- hallucinations*	0.02	0.13	0.22	0.73	-2.04	0.04
*NPI- agitation*	2.09	3.24	0.51	1.08	3.43	< 0.001
*NPI- dysphoria*	1.97	2.75	0.76	1.41	2.89	0.01
*NPI- anxiety*	2.09	3.32	0.62	1.56	2.97	0.01
*NPI- euphoria*	0.19	0.94	0.04	0.18	1.18	0.24
*NPI- apathy*	2.21	3.83	0.58	1.53	2.30	0.01
*NPI- disinhibition*	0.76	2.03	0.27	1.19	1.53	0.12
*NPI- irritability*	1.90	2.83	0.64	1.49	2.93	0.01
*NPI- motor disturbances*	0.57	1.75	0.00	0.00	2.39	0.02
*NPI- sleep disturbances*	0.79	2.11	0.05	0.40	2.54	0.01
*NPI- food issues*	1.05	2.46	0.15	0.62	2.65	0.01
QoL-AD	29.59	9.45	25.09	8.68	2.67	0.01
Loneliness (total score)	15.81	5.11	16.82	4.05	-1.15	0.25
Social loneliness	7.36	2.88	8.38	2.54	-1.98	0.04
Emotional loneliness	8.45	3.01	8.44	2.20	0.02	0.98

NPI: NeuroPsychiatric Inventory; QoL-AD: Quality of Life - Alzheimer’s Disease scale

The correlations between the measures of interest by dementia severity group are shown in Tables S1 and S2, and the results of the hierarchical regression analyses in Tables 2, 3, 4 and 5.

**Table 2:** Hierarchical regression analyses for people with mild dementia, with age, education, gender (step 1), depression (Cornell score, step 2) and total Loneliness score (step 3) as predictors for the outcomes of interest. R^2^, ΔR^2^, and standardized β concern each step, F concerns the final model

	NLT			NPI-total score				NPI-dysphoria			QoL-AD			
	Model 1	Model 2	Model 3		Model 1	Model 2	Model 3	Model 1	Model 2	Model 3	Model 1	Model 2	Model 3	
	β	β	β		β	β	β	β	β	β	β	β	β	
Age	-0.35**	-0.26*	-0.21		-0.54***	-0.29**	-0.26**	-0.50***	-0.33***	-0.42***	-0.12	-0.10	0.08	
Education	0.39*	0.37**	0.35**		0.05	0.003	-0.01	0.15	0.11	0.15	0.03	0.03	-0.04	
Gender^	0.32*	0.28*	0.30*		0.03	-0.08	-0.07	0.17	0.09	0.05	-0.06	-0.06	0.01	
Cornell		0.23	0.22			0.67***	0.67***		0.45***	0.46***		0.04	0.03	
Loneliness			-0.17				-0.09			0.26*			-0.58***	
R2	0.30***	0.35***	0.37***		0.30***	0.69***	0.70***	0.27***	0.45***	0.51***	0.02	0.03	0.30**	
ΔR2	0.30***	0.05	0.02		0.30***	0.39***	0.01	0.27***	0.18***	0.06*	0.02	0.01	0.27***	
	*F*_(5,52)_ = 6.15, *p* < .001			* F*_(5,52)_ = 24.92, *p* < .001				* F*_(5,52)_ = 10.71, *p* < .001			* F*_(5,52)_ = 4.55, *p* = .002			

*p < .05; **p < .01; ***p < .001; ^Gender was a dichotomous variable (0 = male; 1 = female). NLT: Narrative Language Test; NPI: NeuroPsychiatric Inventory; QoL-AD: Quality of Life - Alzheimer’s Disease scale

**Table 3:** Hierarchical regression analyses for people with moderate dementia, with age, education, gender (step 1), depression (Cornell score, step 2) and total Loneliness score (step 3) as predictors for the outcomes of interest. R^2^, ΔR^2^, and standardized β concern each step, F concerns the final model

	NLT			NPI-total score			NPI-dysphoria			QoL-AD			
	Model 1	Model 2	Model 3	Model 1	Model 2	Model 3	Model 1	Model 2	Model 3	Model 1	Model 2	Model 3	
	β	β	β	β	β	β	β	β	β	β	β	β	
Age	-0.05	0.01	-0.002	-0.31*	-0.19	-0.19	-0.12	-0.004	0.003	-0.42**	-0.42**	-0.44**	
Education	0.08	0.05	-0.002	0.16	0.10	0.11	-0.09	-0.15	-0.11	-0.13	-0.13	-0.22	
Gender^	0.19	0.14	0.11	0.05	-0.02	-0.01	-0.00	-0.08	-0.06	0.16	0.16	0.10	
Cornell		0.31*	0.35*		0.61***	0.60***		0.63***	0.60***		-0.01	0.05	
Loneliness			-0.28*			0.05			0.23*			-0.46***	
R2	0.05	0.14	0.22*	0.16*	0.51***	0.51***	0.02	0.39***	0.44***	0.16*	0.16	0.37***	
ΔR2	0.05	0.09*	0.08*	0.16*	0.35***	0.003	0.02	0.38***	0.05*	0.16*	0.00	0.20***	
	*F*_(5,49)_ = 2.76, *p* = .03			* F*_(5,49)_ = 10.31, *p* < .001			* F*_(5,49)_ = 7.80, *p* < .001			* F*_(5,49)_ = 5.68, *p* < .001			

*p < .05; **p < .01; ***p < .001; ^Gender was a dichotomous variable (0 = male; 1 = female). NLT: Narrative Language Test; NPI: NeuroPsychiatric Inventory; QoL-AD: Quality of Life - Alzheimer’s Disease scale

**Table 4:** Hierarchical regression analyses for people with mild dementia, with age, education, gender (step 1), depression (Cornell score, step 2) and scores on Social and Emotional Loneliness subscales (step 3) as predictors for the outcomes of interest. R^2^, ΔR^2^, and standardized β concern each step, F concerns the final model

	NLT			NPI-total score			NPI-dysphoria			QoL-AD		
	Model 1	Model 2	Model 3	Model 1	Model 2	Model 3	Model 1	Model 2	Model 3	Model 1	Model 2	Model 3
	β	β	β	β	β	β	β	β	β	β	β	β
Age	-0.35**	-0.26*	-0.17	-0.54***	-0.29**	-0.26**	-0.50***	-0.33**	-0.44***	-0.11	-0.10	0.12
Education	0.39**	0.37**	0.38**	0.05	0.003	-0.01	0.15	0.11	0.13	0.03	0.03	-0.01
Gender^	0.32*	0.28*	0.28*	0.03	-0.08	-0.07	0.17	0.09	0.07	-0.06	-0.06	-0.01
Cornell		0.23	0.15		0.67***	0.68***		0.45***	0.50***		0.04	-0.03
Social loneliness			-0.31*			-0.02			0.28*			-0.52***
Emotional loneliness			0.10			0.08			0.03			-0.16
R2	0.30***	0.35***	0.40***	0.30***	0.70***	0.71***	0.27***	0.45***	0.52***	0.03	0.03	0.33**
ΔR2	0.30***	0.04	0.06	0.30***	0.40***	0.007	0.27***	0.18***	0.07*	0.02	0.00	0.30***
	*F*_(6,51)_ = 5.81, *p* < .001			* F*_(6,51)_ = 20.42, *p* < .001			* F*_(6,51)_ = 9.19, *p* < .001			* F*_(6,51)_ = 4.21, *p* = .002		

*p < .05; **p < .01; ***p < .001; ^Gender was a dichotomous variable (0 = male; 1 = female). NLT: Narrative Language Test; NPI: NeuroPsychiatric Inventory; QoL-AD: Quality of Life - Alzheimer’s Disease scale

**Table 5:** Hierarchical regression analyses for people with moderate dementia, with age, education, gender (step 1), depression (Cornell score, step 2) and scores on Social and Emotional Loneliness subscales (step 3) as predictors for the outcomes of interest. R^2^, ΔR^2^, and standardized β concern each step, F concerns the final model

	NLT			NPI-total score			NPI-dysphoria			QoL-AD		
	Model 1	Model 2	Model 3	Model 1	Model 2	Model 3	Model 1	Model 2	Model 3	Model 1	Model 2	Model 3
	β	β	β	β	β	β	β	β	β	β	β	β
Age	-0.05	0.01	-0.02	-0.31*	-0.19	-0.18	-0.12	-0.004	0.01	-0.42**	-0.42**	-0.43**
Education	0.08	0.05	-0.06	0.16	0.10	0.12	-0.09	-0.15	-0.09	-0.13	-0.13	-0.21
Gender^	0.19	0.14	0.15	0.05	-0.02	-0.02	-0.01	-0.08	-0.07	0.16	0.16	0.10
Cornell		0.31*	0.24		0.61***	0.63***		0.63***	0.63***		-0.01	0.08
Social Loneliness			-0.49***			0.11			0.23			-0.22
Emotional Loneliness			0.17			-0.05			0.03			-0.32*
R2	0.05	0.14	0.32**	0.16*	0.51***	0.52***	0.02	0.34***	0.38***	0.16*	0.16	0.37***
ΔR2	0.05	0.09*	0.18**	0.16*	0.35***	0.01	0.02	0.38***	0.06	0.16*	0.00	0.21***
	*F*_(6,48)_ = 3.74, *p* = 004			* F*_(6,48)_ = 8.65, p < .001			F_(6,48)_ = 6.55, p < .001			* F*_(6,48)_ = 4.74, p = .001		

*p < .05; **p < .01; ***p < .001; ^Gender was a dichotomous variable (0 = male; 1 = female). NLT: Narrative Language Test; NPI: NeuroPsychiatric Inventory; QoL-AD: Quality of Life - Alzheimer’s Disease scale

#### Total loneliness score as a predictor

*Language skills*. All predictors explained 37% of variance on the NLT for mildly-impaired people with dementia, and the final model was significant, *F*_(5,52)_ = 6.15, *p* < .001. Socio-demographic characteristics accounted for a significant part of variance (30%, p < .001), with younger age, a better education and female gender emerging as significant predictors of better NLT performance (see Table 2). Depression and loneliness did not contribute to explain any additional significant portion of variance on this task (see Table 2), and only a better education (β = 0.35, *p* < .01) and female gender (β = 0.30, *p* < .05) remained significant predictors for better NLT performance in the final model.

For the moderately-impaired people with dementia, all predictors explained 22% of variance in the NLT, and the final model was significant, *F*_(5,49)_ = 2.76, *p* = .03. While socio-demographic characteristics did not account for a significant part of variance (see Table 3), depression contributed to explaining a modest, but significant part of variance (9%, *p* < .05) on this task. Loneliness accounted for an additional significant -though again modest- part of variance (8%, *p* < .05), with worse depression (β = 0.35, *p* < .05) and lower loneliness (β=-0.28, *p* < .05) emerging as significant predictors of better NLT performance in the final model.

*Behavioral and psychological symptoms*. All predictors explained 70% of variance in the total NPI score for mildly-impaired people with dementia, and 50% of variance for moderately-impaired people with dementia, and the final models were significant, *F*_(5,52)_ = 24.92, *p* < .001 and *F*_(5,49)_ = 10.31, *p* < .001 respectively. Socio-demographic characteristics accounted for a significant part of the variance for both mildly-impaired people with dementia (30%, *p* < .001) and moderately-impaired people with dementia (16%, p < .05), with younger age predicting more frequent and severe BPS in both groups (β=-0.54, *p* < .001 and β=-0.31, *p* < .05, respectively). Depression accounted for an additional significant portion of variance in the NPI total score in both groups (39%, *p* < .001 and 35%, *p* < .001, respectively), with worse depression predicting more frequent and severe BPS for both mildly-impaired people with dementia (β = 0.67, *p* < .05) and moderately-impaired people with dementia (β = 0.61, *p* < .05). Loneliness did not contribute to explain an additional portion of variance in either group (see Tables 2 and 3). Younger age (β=-0.26, *p* < .01) and worse depression (β = 0.67, *p* < .001) in the mildly-impaired people with dementia, and worse depression only (β = 0.60, *p* < .001) in the moderately-impaired ones remained the only significant predictors of more frequent and severe BPS in the final models.

Looking at the specific symptoms examined by the NPI^2^, loneliness only contributed to explain an additional significant -though modest- part of variance on the dysphoria subscale, for both the mildly-impaired group (6%, p < .05) and the moderately-impaired group (5%, p < .05). For the former, younger age, worse depression and loneliness emerged as significant predictors of dysphoria; for the latter, only worse depression and loneliness were significant predictors of dysphoria in the final models, which were significant, *F*_(5,52)_ = 10.71, *p* < .001 and *F*_(5,49)_ = 7.80, *p* < .001 respectively (see Tables 2 and 3 for details).

*Quality of life.* All predictors explained 30% of variance in the QoL-AD scores for the mildly-impaired people with dementia, and 37% for the other group, and the models were significant, *F*_(5,52)_ = 4.55, *p* = .002 and *F*_(5,49)_ = 5.68, *p* < .001 respectively. Socio-demographic characteristics and depression did not account for a significant part of variance in the former group (see Table 2), and loneliness contributed to explaining an additional significant portion of variance (27%, *p* < .001) on the QoL-AD scores, with less loneliness (β=-0.58, *p* < .001) predicting higher QoL-AD scores. For the moderately-impaired group, socio-demographic characteristics accounted for a significant part of variance (16%, *p* < .05) on this scale, with younger age (β=-0.42, *p* < .01) emerging as a significant predictor of a better QoL. Depression did not account for an additional significant portion of variance on this scale (see Table 3), while loneliness contributed to explaining an additional significant portion of variance (20%, *p* < .001) on the QoL-AD scores. Younger age (β=-0.44, *p* < .01) and less loneliness (β=-0.46, *p* < .001) were significant predictors of better perceived QoL in the final model (see Table 3).

#### Social and emotional loneliness subscales as predictors

*Language skills*. All predictors explained 40% of variance in the NLT scores for the mildly-impaired people with dementia, and 32% for the moderately-impaired group, and the final models were significant, *F*_(6,51)_ = 5.81, *p* < .001 and *F*_(6,48)_ = 3.74, *p* = 004 respectively. In the former, the two loneliness subscales did not explain any additional significant portion of variance when added in Step 3 as predictors (see Table 4), but less social loneliness (β=-0.31, *p* < .05), along with a better education (β = 0.38, *p* < .01) and female gender (β = 0.28, *p* < .05) emerged as significant predictors of a better NLT performance in the final model. As for the moderately-impaired people with dementia, the two loneliness subscales contributed to explaining an additional significant portion of variance in this task (18%, *p* < .01) when added in Step 3 as predictors, with only less social loneliness (β=-0.49, *p* < .001) predicting a better performance on the NLT in the final model (see Table 5).

*Behavioral and psychological symptoms*. For the mildly-impaired group, adding the Social and Emotional Loneliness subscales as predictors did not change the results for the total NPI score (see Tables 4 and 5), but did confirm that loneliness explained a modest but significant additional part of variance in dysphoria symptoms (7%), with more social loneliness emerging as a significant predictor of this symptoms in this group, along with younger age and worse depression (see Table 4). For the moderately-impaired group, the two loneliness subscales did not account for any significant additional variance, and worse depression remained the only significant predictor of dysphoria symptoms (see Table 5).

*Quality of life*. All predictors explained 33% of variance in the QoL-AD scores for the mildly-impaired people with dementia, and 37% for the moderately-impaired group, and the final models were significant, *F*_(6,51)_ = 4.21, *p* = .002 and *F*_(6,48)_ = 4.74, p = .001 respectively. Loneliness was confirmed to account for an additional significant portion of variance on the QoL-AD scores for both mildly-impaired people with dementia (30%, *p* < .001) and moderately-impaired ones (21%, *p* < .001). Less social loneliness (β=-0.52, *p* < .001) emerged as the only significant predictor of higher QoL-AD scores for the mildly impaired, while less emotional loneliness (β=-0.32, *p* < .05), with younger age (β=-0.43, *p* < .01), were significant predictors of better perceived QoL for the moderately impaired in the final models (see Tables 4 and 5).

## Discussion

This study examined the relationship between loneliness and its social and emotional facets, and language skills, BPS (as assessed through the NPI), and QoL in people with dementia, also innovatively considering dementia severity (mild vs. moderate).

In line with the literature [50], and our hypotheses, the mildly-impaired people with dementia outperformed the moderately-impaired group in terms of language skills (as measured by the NLT), had higher NPI scores, more severe depression, and a reportedly better QoL. The two groups did not differ in terms of overall loneliness or its emotional facet, but mildly-impaired people with dementia reported less social loneliness than moderately-impaired ones. Such findings suggest and confirm that people with dementia generally feel lonely [32, 33] and that, as dementia progresses, older adults are more likely to experience a decline in social network [36]. They also seem to indicate that, regardless of illness stage, people with dementia more likely experience emotional loneliness, rather than the social one. These results are only partially in line with the only other previous study examining social and emotional loneliness in people with dementia, which reported social loneliness increasing and emotional loneliness decreasing with dementia progression [34]. However, in Holmen’s study [34], unlike here, loneliness’s facets were examined by using a single question each. Therefore, it seems important to recognize the complexity of loneliness [20, 31, 66], exploring it not only as a global construct but also examining its different dimensions.

Results from our regression analyses then allowed us to clarify the relationship between loneliness and language skills, BPS and QoL in people with dementia, also considering the role of socio-demographic characteristics and mood. They revealed a different pattern of results depending on dementia stage, but also on the facets of loneliness that we considered.

Globally, loneliness significantly contributed to explain language skills and QoL, but not total NPI scores, in line with previous evidence [39, 40], as socio-demographic characteristics and depression accounted for the observed variance in frequency and severity of BPS. However, loneliness additionally contributed -though slightly- to explain variance in dysphoric symptoms (a state of general malaise, unhappiness and discontent, sometimes associated with depression) as assessed through the NPI subscale. In other words, also loneliness seems to contribute –though modestly– to exacerbate the dissatisfaction and sadness characteristic of dysphoria, despite the increasingly disturbed cognition or other functional limitations caused by dementia.

Interestingly, in line with our expectations, loneliness uniquely contributed to explain the observed variance in QoL in mildly-impaired people with dementia, and was associated with a better QoL in the moderately impaired, along with younger age (possibly affecting the health-related aspect of QoL). Such a clear, major influence of loneliness on QoL extends previous evidence [38, 41] to patients with mild or moderate dementia. It also confirms preliminary data on the relationship between loneliness and dementia indicators [38, 41] calling upon the need of exploring loneliness when the complex construct of QoL in dementia is the target of clinical assessments and interventions [67, 68].

It is worth adding that, although more severe depression (but not loneliness) predicted more frequent and severe BPS in both mild (along with age) and moderate dementia, depression did not affect QoL in either group. In short, such a pattern of results indicated that, as previously demonstrated [39, 40], loneliness did not influence the severity of BPS, but had a unique impact on QoL (not mediated by psychological symptoms of dementia), confirming that loneliness is distinct from depression in people with mild-to-moderate dementia [69].

Lower loneliness also additionally contributed to predict better language skills in moderately-impaired people with dementia, albeit only modestly. These findings innovatively suggest that when the language skills needed in social relationships are better preserved, people with dementia are more satisfied with the frequency and closeness of their relationships. Since this association was only seen in our moderately-impaired sample, efforts should be made to support patients’ communication skills in order to contain their social isolation, and sustain their QoL, as their mental capacities decline.

Interestingly, considering the social and emotional facets of loneliness enabled us to obtain a more detailed and specific picture of the relationship between loneliness and language functioning, BPS and QoL in people with dementia.

In particular, we found the social facet of loneliness (and not loneliness as a unitary construct) to predict language skills in both groups of more or less severely impaired people with dementia. As a rich social network may give the adequate social support and engagement through person-to-person contact, cognition –at least language functioning as measured here- could be beneficially affected via either physiological or psychological pathways [70, 71]. Satisfying and significant social interactions, as well as their maintenance, can positively influence the stress-related responses known to heighten the risk of developing dementia. On the other hand, an increased number of meaningful social interactions may offer the adequate level of mental -and physical- stimulation to preserve such an important cognitive function [14, 72]. Focusing on reducing social loneliness, by improving social engagement and increasing opportunities for mental stimulation might therefore be important for preserving the cognitive and, in particular, communication abilities that people with dementia need to engage, reciprocate and maintain social relationships; and this would particularly benefit the more impaired [43, 54, 68].

Social loneliness was also the only facet that additionally contributed to predict (though only modestly compared with age and depression) more frequent and severe dysphoria symptoms. This only applied to mildly-impaired people with dementia, with a better-preserved cognitive profile, possibly because they are more aware of their condition and therefore more affected by the difficulty they experience with maintaining meaningful social relationships. This could lead them to perceive their social support network as unsatisfactory and inadequate, and thus explain their more frequent and severe feelings of unease and dissatisfaction characteristic of dysphoria. The two facets of loneliness did not predict dysphoria symptoms in moderately-impaired people with dementia, however; depression was the only significant predictor in their case. The modest part of variance explained (as also emerged for the total loneliness score) means that these results should be taken with caution, however. It is worth mentioning that our sample displayed overall low NPI scores, and anyone with severe psychiatric disturbances was excluded. Therefore, further research will be needed to clarify the complex interplay between depression, dysphoria, and loneliness, and the latter link with other BPS in people with dementia, also in relation to dementia severity.

Intriguingly, considering the social and emotional loneliness facets separately afforded a clearer picture also of how the different features of these subjective feelings affected the QoL of people with dementia, and how their role differed, depending on the dementia stage considered. Social loneliness accounted for QoL scores in mildly-impaired people with dementia, whereas emotional loneliness (alongside younger age) emerged as a significant predictor of QoL in the moderately-impaired group. This pattern of results again underscores the complex relationship between cognitive functioning, QoL and the distressing feeling that occurs when people’s social and emotional needs are not met by the size and quality of their existing social relationships. In people with dementia with a less impaired cognitive profile, QoL is affected by the perceived feeling of missing social contacts and degree of social support. On the other hand, for younger people with dementia coping with a more severe stage of the disorder, their perceived QoL depends more on how they perceive the quality of close and intimate relationships: they need to feel they have a network of contacts capable of meeting other than their instrumental needs, but also their need for reciprocal, satisfying and emotionally-supportive relationships [37, 53–55].

Despite these interesting findings, some limitations of this study deserve to be acknowledged. First, other characteristics relating to dementia (e.g., age at onset of the disorder and its duration, use of medication), and to people’s social networks (e.g., frequency of contacts with family/friends, size of social network) -not available here- would need to be taken into account to help disentangle the association between loneliness and cognitive, BPS and QoL in people with dementia. Also, though we attempted to account for dementia severity, we focused only on the mild and moderate stages of the disorder, and the cross-sectional nature of the study prevented us from ascertaining any causal relationships between loneliness and its facets, and disease progress.

Taken together, our results nonetheless innovatively suggest that considering loneliness as a multidimensional construct -at least with its social and emotional facets as here- can be very informative. Loneliness and its facets have a strong impact on how people with dementia perceive their QoL, and also influence (to a lesser extent) their language skills and dysphoria. Their role varies, however, depending on a crucial individual characteristic, the dementia severity. From a clinical and applied perspectives, these results underscore the importance of systematically including the assessment of loneliness and its social and emotional facets in people with dementia to account for their different associations with cognitive functioning and -specific- psychological and behavioral symptoms considering the severity of dementia, and develop effective interventions to counter it.

## Electronic supplementary material

Below is the link to the electronic supplementary material.

Supplementary Material 1

## Data Availability

The data that support the findings of this study are available from the corresponding authors on reasonable request.

## Footnotes

1 This value could be considered as adequate given the number of items (3) included in the scale [73].

2 None of the predictors contributed to explaining scores for *delirium, hallucinations, euphoria, sleep, motor* or *food disturbances* in either group. Depression was the only significant predictor for: *agitation* in both mild (β = 0.64, *p* < .001; R2 = 0.49, *F*_(5,52)_ = 9.93, *p* < .001) and moderate (β = 0.68, *p* < .001; R2 = 0.51, *F*_(5,49)_ = 2.97, *p* = .02) dementia; *anxiety* in both mild (β = 0.68, *p* < .001; R2 = 0.51, *F*_(5,52)_ = 10.79, *p* < .001) and moderate (β = 0.48, *p* = .001; R2 = 0.47, *F*_(5,49)_ = 8.74, *p* < .001) dementia; (iii) *irritability* in both mild (β = 0.52, *p* < .001; R2 = 0.40, *F*_(5,52)_ = 7.08, *p* < .001) and moderate (β = 0.29, *p* = .03; R2 = 0.19, *F*_(5,49)_ = 2.43, *p* = .04) dementia; *disinhibition* only in moderate dementia (β = 0.29, *p* = .03; R2 = 0.21, *F*_(5,49)_ = 2.55, *p* = .03). Age (β=-0.27, *p* = .04) and depression (β = 0.39, *p* = .002) both emerged as significant predictors for *apathy*, but only in mild dementia (R2 = 0.37, *F*_(5,52)_ = 6.09, *p* < .001). Neither loneliness nor its social and emotional facets contributed to explaining scores for these symptoms when added in Step 3 as predictors.
