# Supplementary material for: Individual differences among older adults with mild and moderate dementia in social and emotional loneliness and their associations with cognitive and psychological functioning
Source: BMC Geriatr. 2022 Nov 15;22:859. doi: 10.1186/s12877-022-03517-2 (PMC9667624; doi:10.1186/s12877-022-03517-2)
Supplement: Supplementary file 1 — Supplementary Material 1 [file 12877_2022_3517_MOESM1_ESM.docx]

Individual differences among older adults with mild and moderate dementia in social and emotional loneliness and their associations with cognitive and psychological functioning

Supplementary materials

Table S1. Matrix of correlations between the measures of interest for people with mild dementia.

|  | 1 | 2 | 3 | 4 | 5 | 6 | 7 | 8 | 9 | 10 | 11 | 12 | 13 | 14 | 15 | 16 | 17 | 18 | 19 | 20 | 21 |
| --- | --- | --- | --- | --- | --- | --- | --- | --- | --- | --- | --- | --- | --- | --- | --- | --- | --- | --- | --- | --- | --- |
| 1. Social loneliness | -- |  |  |  |  |  |  |  |  |  |  |  |  |  |  |  |  |  |  |  |  |
| 2. Emotional loneliness | .50** | -- |  |  |  |  |  |  |  |  |  |  |  |  |  |  |  |  |  |  |  |
| 3. Loneliness (total score) | .86** | .87** | -- |  |  |  |  |  |  |  |  |  |  |  |  |  |  |  |  |  |  |
| 4. Age | .45** | .26 | .41** | -- |  |  |  |  |  |  |  |  |  |  |  |  |  |  |  |  |  |
| 5. Education | -.17 | -.29* | -.26* | -.28* | -- |  |  |  |  |  |  |  |  |  |  |  |  |  |  |  |  |
| 6. Gender^ | .18 | .30* | .28* | .35** | -.31* | -- |  |  |  |  |  |  |  |  |  |  |  |  |  |  |  |
| 7. NLT | -.41** | -.12 | -.30* | -.35** | .39** | .08 | -- |  |  |  |  |  |  |  |  |  |  |  |  |  |  |
| 8. Cornell score | -.32* | .07 | -.14 | -.33* | .13 | .03 | .37** | -- |  |  |  |  |  |  |  |  |  |  |  |  |  |
| *9. NPI- delirium* | -.03 | .26* | .14 | -.07 | .10 | .16 | .17 | .31* | -- |  |  |  |  |  |  |  |  |  |  |  |  |
| *10. NPI- hallucinations* | .12 | .29* | .24 | .09 | -.16 | .10 | .09 | .04 | -.03 | -- |  |  |  |  |  |  |  |  |  |  |  |
| *11. NPI- agitation* | -.38** | -.03 | -.23 | -.30* | -.02 | -.15 | .36** | .64** | .06 | .16 | -- |  |  |  |  |  |  |  |  |  |  |
| *12. NPI- dysphoria* | -.08 | .08 | .01 | -.48** | .24 | -.05 | .38** | .58** | .08 | -.10 | .29* | -- |  |  |  |  |  |  |  |  |  |
| *13. NPI- anxiety* | -.35** | -.08 | -.25 | -.24 | .03 | .01 | .33* | .68** | .19 | -.08 | .57** | .51** | -- |  |  |  |  |  |  |  |  |
| *14. NPI- euphoria* | -.11 | .05 | -.03 | .01 | .10 | -.09 | .16 | .06 | -.04 | .12 | .38** | -.12 | .01 | -- |  |  |  |  |  |  |  |
| *15. NPI- apathy* | -.23 | -.20 | -.25 | -.46** | .26* | -.17 | .24 | .50** | .34** | -.08 | .18 | .47** | .38** | -.10 | -- |  |  |  |  |  |  |
| *16. NPI- disinhibition* | -.13 | .01 | -.07 | -.33* | .15 | -.12 | .25 | .15 | .24 | .21 | .30* | .02 | -.01 | .43** | .15 | -- |  |  |  |  |  |
| *17. NPI- irritability* | -.35** | -.09 | -.25 | -.39** | .13 | -.13 | .43** | .58** | .09 | .19 | .53** | .38** | .59** | .14 | .49** | .21 | -- |  |  |  |  |
| *18. NPI- motor disturbances* | -.25* | -.33** | -.34** | -.22 | -.02 | -.03 | .19 | .17 | -.07 | -.04 | .28* | .06 | .25 | .06 | .12 | .25 | .27* | -- |  |  |  |
| *19. NPI- sleep disturbances* | -.23 | .04 | -.11 | -.20 | .06 | -.11 | .30* | .38** | -.03 | -.05 | .46** | .32* | .34** | .06 | .07 | -.05 | .37** | -.12 | -- |  |  |
| *20. NPI- food issues* | -.28* | -.25 | -.31* | -.38** | .20 | -.19 | .22 | .26* | .40** | -.06 | .15 | .26* | .08 | .27* | .57** | .23 | .30* | .19 | .07 | -- |  |
| 21 NPI (total score) | -.42** | -.13 | -.31* | -.54** | .20 | -.17 | .50** | .77** | .29* | .04 | .71** | .62** | .72** | .24 | .68** | .37** | .78** | .36** | .44** | .54** | -- |
| 22. QoL-AD | -.54** | -.40** | -.54** | -.15 | .09 | -.11 | .22 | .08 | .03 | .09 | .18 | -.19 | .18 | .14 | .24 | .08 | .26* | .14 | .07 | .29* | .25 |

*p<.05; **p<.01; ^Gender was a dichotomous variable (0=male; 1=female). NLT: Narrative Language Test; NPI: NeuroPsychiatric Inventory; QoL-AD: Quality of Life - Alzheimer’s Disease scale.

Table S2. Matrix of correlations between the measures of interest for people with moderate dementia.

|  | 1 | 2 | 3 | 4 | 5 | 6 | 7 | 8 | 9 | 10 | 11 | 12 | 13 | 14 | 15 | 16 | 17 | 18 | 19 | 20 |
| --- | --- | --- | --- | --- | --- | --- | --- | --- | --- | --- | --- | --- | --- | --- | --- | --- | --- | --- | --- | --- |
| 1. Social loneliness | -- |  |  |  |  |  |  |  |  |  |  |  |  |  |  |  |  |  |  |  |
| 2. Emotional loneliness | .44** | -- |  |  |  |  |  |  |  |  |  |  |  |  |  |  |  |  |  |  |
| 3. Loneliness (total score) | .87** | .82** | -- |  |  |  |  |  |  |  |  |  |  |  |  |  |  |  |  |  |
| 4. Age | .05 | -.04 | .01 | -- |  |  |  |  |  |  |  |  |  |  |  |  |  |  |  |  |
| 5. Education | -.25 | -.01 | -.16 | -.40** | -- |  |  |  |  |  |  |  |  |  |  |  |  |  |  |  |
| 6. Gender^ | -.05 | -.12 | -.10 | .10 | -.02 | -- |  |  |  |  |  |  |  |  |  |  |  |  |  |  |
| 7. NLT | -.42** | .01 | -.26* | -.07 | .11 | .18 | -- |  |  |  |  |  |  |  |  |  |  |  |  |  |
| 8. Cornell | -.08 | .26* | .10 | -.22 | .18 | .11 | .34* | -- |  |  |  |  |  |  |  |  |  |  |  |  |
| *9. NPI- delirium* | .03 | .28* | .18 | -.01 | -.08 | -.08 | -.09 | .16 | -- |  |  |  |  |  |  |  |  |  |  |  |
| *10. NPI- hallucinations* | -.07 | .12 | .03 | -.05 | -.03 | .07 | .10 | .23 | .23 | -- |  |  |  |  |  |  |  |  |  |  |
| *11. NPI- agitation* | .06 | .05 | .06 | .02 | -.02 | -.13 | .08 | .42** | .27 | -.05 | -- |  |  |  |  |  |  |  |  |  |
| *12. NPI- dysphoria* | .22 | .31* | .31* | -.09 | -.04 | -.02 | .10 | .60** | .11 | .12 | .08 | -- |  |  |  |  |  |  |  |  |
| *13. NPI- anxiety* | -.06 | .20 | .07 | -.44** | .37** | .14 | .22 | .57** | .24 | -.12 | .13 | .36** | -- |  |  |  |  |  |  |  |
| *14. NPI- euphoria* | .01 | .09 | .06 | -.13 | -.01 | .12 | .19 | .01 | -.09 | -.06 | -.09 | .03 | -.08 | -- |  |  |  |  |  |  |
| *15. NPI- apathy* | -.001 | -.11 | -.06 | -.23 | .19 | -.01 | -.10 | .02 | .04 | -.05 | -.07 | .15 | -.08 | .43** | -- |  |  |  |  |  |
| *16. NPI- disinhibition* | -.01 | -.03 | -.02 | -.30* | .26 | .07 | -.04 | .36** | .18 | -.07 | .30* | .35** | .51** | -.05 | -.03 | -- |  |  |  |  |
| *17. NPI- irritability* | -.07 | -.05 | .07 | -.28* | .29* | -.01 | .02 | .34** | .27* | .01 | .53** | .17 | .34* | .24 | .21 | .70** | -- |  |  |  |
| *18.. NPI- sleep disturbances* | -.02 | .16 | .07 | .02 | -.01 | -.22 | -.03 | .17 | .27* | -.04 | -.06 | .22 | .12 | -.03 | .13 | -.03 | -.06 | -- |  |  |
| *19.. NPI- food issues* | -.26 | .03 | -.14 | -.17 | .26* | .15 | .38** | .35** | .08 | .01 | .14 | .04 | .57** | -.05 | -.01 | .02 | .04 | -.03 | -- |  |
| 20. NPI (total score) | -.003 | .17 | .09 | -.37** | .28* | .02 | .12 | .67** | .46** | .12 | .47** | .58** | .66** | .15 | .34** | .69** | .75** | .19 | .32* | -- |
| 21. QoL-AD | -.35** | -.39** | -.43** | -.35** | .04 | .12 | .14 | .08 | -.08 | -.01 | .19 | -.35** | .02 | .26 | .08 | .14 | .28* | -.03 | .03 | .07 |

*p<.05; **p<.01; ^Gender was a dichotomous variable (0=male; 1=female). NLT: Narrative Language Test; NPI: NeuroPsychiatric Inventory; QoL-AD: Quality of Life - Alzheimer’s Disease scale. Note: since people with moderate dementia did not have motor disturbances, this NPI subscale was not considered
